# Echoes of Muscle Aging: The Emerging Role of Shear Wave Elastography in Sarcopenia Diagnosis

**DOI:** 10.3390/diagnostics15192495

**Published:** 2025-09-30

**Authors:** Linda Galasso, Federica Vitale, Manuela Pietramale, Giorgio Esposto, Raffaele Borriello, Irene Mignini, Antonio Gasbarrini, Maria Elena Ainora, Maria Assunta Zocco

**Affiliations:** CEMAD Digestive Disease Center, Fondazione Policlinico Universitario Agostino Gemelli IRCCS, Catholic University of Rome, 00168 Rome, Italy; linda.galasso@guest.policlinicogemelli.it (L.G.); federica.vitale@guest.policlinicogemelli.it (F.V.); manuela.pietramale01@icatt.it (M.P.); giorgio.esposto@guest.policlinicogemelli.it (G.E.); raffaele.borriello@unicatt.it (R.B.); irene.mignini@guest.policlinicogemelli.it (I.M.); antonio.gasbarrini@unicatt.it (A.G.); mariaelena.ainora@policlinicogemelli.it (M.E.A.)

**Keywords:** shear wave elastography (SWE), sarcopenia

## Abstract

Sarcopenia, a progressive age-related loss of skeletal muscle mass, strength, and function, is a major contributor to disability, reduced quality of life, and mortality in older adults. While current diagnostic approaches, such as dual-energy X-ray absorptiometry (DXA), bioelectrical impedance analysis (BIA), magnetic resonance imaging (MRI), and computed tomography (CT), are widely used to assess muscle mass, they have limitations in detecting early qualitative changes in muscle architecture and composition. Shear Wave Elastography (SWE), an ultrasound-based technique that quantifies tissue stiffness, has emerged as a promising tool to evaluate both muscle quantity and quality in a non-invasive, portable, and reproducible manner. Studies suggest that SWE can detect alterations in muscle mechanical properties associated with sarcopenia, providing complementary information to traditional morphometric assessments. Preliminary evidence indicates its good reproducibility, feasibility in various clinical settings, and potential for integration into routine evaluations. This narrative review summarizes current evidence on the use of SWE for the assessment of sarcopenia across diverse populations.

## 1. Introduction

Sarcopenia, first described by Irwin Rosenberg in 1989, refers to the progressive loss of skeletal muscle mass associated with aging [1]. Over time, the definition has expanded to include declines in muscle strength and physical performance, and in 2016, the World Health Organization officially classified it as a disease (ICD-10-CM code M62.84) [2,3]. Sarcopenia is now widely recognized as a significant geriatric syndrome linked to functional decline, disability, reduced quality of life, and increased mortality [2]. Its global prevalence ranges from 10–20% among older adults, depending on diagnostic criteria used [4,5,6]. Several expert panels, including European Working Group on Sarcopenia in Older People (EWGSOP) [7], the International Working Group on Sarcopenia (IWGS) [8], the Asian Working Group for Sarcopenia (AWGS) [9], the Foundation for the National Institutes of Health (FNIH) [10], and the Sarcopenia Definition and Outcomes Consortium (SDOC) [11], have established diagnostic frameworks and consensus criteria. The original EWGSOP classification (2010) categorized sarcopenia into stages of severity, while the 2018 EWGSOP2 update emphasized low muscle strength as the primary indicator, with reduced muscle mass confirming the diagnosis and impaired physical performance identifying severe cases [7]. The SDOC further highlighted low grip strength and slow gait speed as strong predictors of adverse outcomes, noting that lean mass measured by DXA lacked independent predictive value after adjusting for body size [11]. In the context of early detection, EWGSOP2 recommends the SARC-F questionnaire, a brief, self-reported tool evaluating difficulties in strength, mobility, and fall history, as a screening measure, particularly in community-dwelling older adults [12]. Diagnostic confirmation, however, still relies on objective tools. DXA remains widely accepted as a reference method for assessing muscle mass due to its low radiation exposure, cost-effectiveness, and rapid acquisition. Despite these advantages, DXA is non-portable and susceptible to error in patients with altered hydration status, as it cannot differentiate between water and lean soft tissue. Furthermore, DXA lacks the ability to assess muscle quality, particularly regarding fat infiltration and microstructural changes. Alternative modalities such as Bioelectrical Impedance Analysis (BIA) offer portability and affordability but provide only indirect estimates of muscle mass based on tissue conductivity. Their accuracy is significantly influenced by factors such as age, hydration, ethnicity, and comorbidities. MRI and CT offer precise assessments of muscle quantity and quality, but their high cost, operational complexity, and limited accessibility, along with radiation exposure in the case of CT, restrict their use primarily to research settings [13]. In addition, the provider of harmonized cut-off values across imaging modalities limits comparability and contributes to variability in prevalence estimates across studies [12]. Given these limitations, there is a critical need for accessible and accurate tools capable of evaluating not just muscle quantity, but also muscle quality. Ultrasound imaging has emerged as a valuable option due to its portability, safety, and cost-effectiveness, with good agreement with reference methods. However, conventional ultrasound faces challenges in clearly differentiating muscle from fat and is susceptible to operator-dependent variability [13,14]. To address these limitations, Shear Wave Elastography (SWE) has emerged as an innovative and clinically promising tool for the evaluation of skeletal muscle [15].

## 2. Methods

This narrative review discusses the emerging role of SWE in the diagnostic work-up of sarcopenia, highlighting its potential to bridge current diagnostic gaps and enhance our ability to assess, monitor, and ultimately treat this increasingly prevalent age-related condition. The literature search was conducted using PubMed and included all scientific studies related to the use of SWE in sarcopenia.

## 3. Muscle Stiffness via SWE as a Window into Sarcopenia

Before describing SWE, it is important to first address the concept of muscle stiffness. Age-related deterioration of skeletal muscle involves intertwined declines in mass, strength, and quality, shaped by genetic, hormonal, metabolic, and lifestyle determinants [16]. Although the progressive loss of fast-twitch fibers starting from around age 30 and leading to a 30–40% reduction in total volume and lean mass by age 80 [17,18], remains a hallmark of sarcopenia, this structural atrophy alone does not fully explain functional deficits. Uneven atrophy across muscle groups, exacerbated by endocrine changes and physical inactivity [19], interacts with neuromuscular impairments such as reduced motor unit recruitment, slowed conduction, and degraded neuromuscular junction function (NMJ), all of which compromise contraction velocity and rate of force development [20,21]. Contemporary definitions therefore emphasize muscle quality as a decisive factor in the functional expression of sarcopenia [22,23,24]. Structural alterations, myosteatosis [25,26], NMJ degeneration [27], mitochondrial dysfunction with heightened oxidative stress [28], combined with changes in architecture, ultrastructure, and composition [29,30,31] collectively contributed to the erosion of muscle performance per unit mass. The challenge remains that conventional diagnostic tools such as DEXA or CT can measure mass and detect fat infiltration, but they cannot adequately capture early functional decline [32,33]. In this context, biomechanical profiling has gained importance. Properties such as stiffness, elasticity, viscoelasticity, and contractile dynamics [34] change early in the degenerative process, often before overt mass loss. Reduced elasticity from titin isoform shifts, advanced glycation end products accumulation, and altered hydration [35], together with age-related changes in viscoelasticity [36] and contractile dynamics [37], signal subtle impairments in load adaptation, force transmission, and postural control that precede clinical weakness. Among these, muscle stiffness has emerged as a particularly sensitive early biomarker [38], reflecting both intrinsic (fiber-type shifts) and extrinsic (connective tissue and fat infiltration) drivers of reduced adaptability and increased injury risk. It is precisely regarding muscle stiffness that SWE plays a critical role [39].

## 4. Principles of Shear Wave Elastography (SWE)

**Figure 1:**
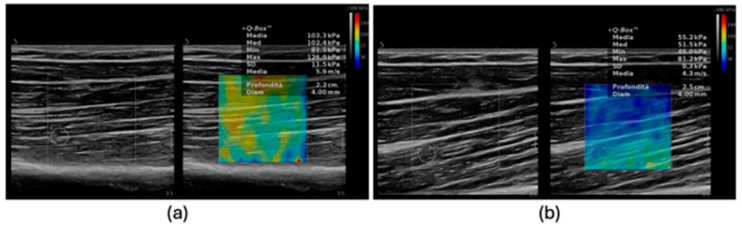
Differences in muscle stiffness assessed by SWE: (**a**) Increased stiffness observed in an elderly patient with myosteatosis and sarcopenia (103.3 kPa). (**b**) Reduced stiffness noted in a younger patient (55.2 kPa). SWE, shear wave elastography; kPa, kilopascal.

SWE is an ultrasound-based elastographic technique that quantitatively measures tissue stiffness by assessing the velocity of shear waves propagating through the tissue [40]. The method employs acoustic radiation force impulse (ARFI) to generate localized, transient displacements within the tissue, which in turn produce shear waves traveling perpendicularly to the ultrasound beam [41]. Using ultrafast imaging (thousands of frames per second), the system tracks shear wave velocity (SWS, m/s), which is directly proportional to the tissue’s mechanical stiffness. SWS can be converted into Young’s modulus (kPa) using the equation E = 3ρc^2^, where E is Young’s modulus, ρ is tissue density (assumed constant), and c is shear wave speed [40]. In musculoskeletal applications, SWE can assess both passive and active mechanical properties of skeletal muscle. Given that muscle stiffness is anisotropic and influenced by factors such as fiber orientation, contraction state, and load, probe alignment with muscle fascicles is crucial. Measurements are typically obtained in longitudinal sections, parallel to the fibers, to minimize variability [15]. Standardization of acquisition parameters, including region of interest size and depth, minimal transducer pressure, and consistent patient posture with full muscle relaxation, is essential to reduce both inter- and intra-operator variability. In the context of sarcopenia, SWE provides additional information on muscle quality alongside conventional B-mode ultrasound metrics such as thickness, echogenicity, and pennation angle [42]. Examinations are performed with the patient in a standardized position (supine for anterior muscles, prone for posterior muscles), using a high-frequency linear transducer (7–15 MHz) placed over the mid-belly of the selected muscle, commonly the quadriceps femoris, in longitudinal or transverse orientation. Minimal pressure is applied to avoid compression artifacts [15]. Once in SWE mode, acquisition settings are optimized for musculoskeletal imaging, shear wave propagation is recorded within a defined region of interest, and the resulting elastographic map (color-coded) reflects the spatial distribution of stiffness (Figure 1) [43]. Mean or median stiffness values are calculated over multiple acquisitions to improve reproducibility. By providing a quantitative, reproducible, and non-invasive biomarker of muscle mechanical properties, SWE represents a promising tool for the diagnosis and monitoring of sarcopenia.

In contrast to DXA and BIA, which estimate muscle quantity, SWE can detect early changes in muscle elasticity associated with fibrosis, fatty infiltration, or altered microstructure, which are not visible morphologically [44]. While MRI and CT remain the gold standards for high-resolution structural assessment, their limitations, including cost, accessibility, and radiation exposure (CT), make SWE a compelling tool for both clinical and longitudinal monitoring [45]. Despite its advantages, SWE is not without technical challenges. Tissue anisotropy, depth-related signal attenuation, and acoustic shadowing from adjacent bone structures can influence the accuracy of stiffness measurements. Additionally, there is currently no universal standard for normal reference values in skeletal muscle, and inter-vendor variability remains an issue.

To improve reproducibility and provide a practical guide for SWE measurements in sarcopenia, we created a checklist of the key steps of the ultrasound methodology (Table 1).

## 5. Clinical Applications of SWE in Sarcopenia: Evidence Across Different Patient Populations

Recent literature underscores the expanding role of SWE as a quantitative, non-invasive modality for assessing muscle quality in sarcopenia. Several studies have evaluated SWE in cohorts with sarcopenia or related conditions, demonstrating muscle-specific alterations in stiffness that correlate with functional decline. Notably, investigations have focused on the quadriceps muscle group, particularly the rectus femoris (RF), and the medial gastrocnemius (GCM), both of which are critical for mobility and strength.

### 5.1. Quantitative Muscle Assessment in Type 2 Diabetes Using SWE: Correlations, Cut-Offs and Clinical Utility

Patients with type 2 diabetes (T2D) are at increased risk of accelerated muscle deterioration due to combined metabolic, inflammatory, and vascular factors [46,47,48]. In recent years, SWE has emerged as a promising imaging modality for the quantitative assessment of skeletal muscle quality in this population.

#### 5.1.1. Evidence from Gastrocnemius Muscle Studies (GCM)

Li An et al. reported significantly reduced shear wave velocity (SWV) in the medial GCM of T2D patients with sarcopenia compared with nonsarcopenic counterparts, with median SWV values markedly lower in sarcopenic individuals (*p* < 0.01) [49]. In the same cohort, SWV measured in the bent GCM demonstrated robust positive correlations with ASMI and handgrip strength (*p* < 0.01). Building on these findings, Li An et al. developed a logistic regression model incorporating body mass index (BMI), ASMI, and SWV values (both in straight and bent muscle states), achieving excellent diagnostic performance, with a sensitivity of 96.7%, a specificity of 82.5%, and an area under the curve (AUC) of 0.946. Similarly, Wei et al. assessed the gastrocnemius muscle in T2D patients and found that sarcopenic individuals had significantly lower muscle thickness, cross-sectional area (CSA), and SWE values than nonsarcopenic individuals (all *p* < 0.05). In their diagnostic model, SWE-based parameters yielded a sensitivity of 81.1%, specificity of 75.0%, and AUC of 0.800 [50]. Moderate correlations were also observed between SWE and both muscle mass (r = 0.26, *p* = 0.002) and strength parameters, further highlighting the utility of evaluating multiple muscle groups in diabetic myopathy, which may present heterogeneously.

#### 5.1.2. Evidence from Rectus Femoris Muscle Studies

Chen et al. investigated the RF in T2D patients using both SWE and conventional ultrasound (thickness and CSA) [51]. Sarcopenic individuals exhibited significantly lower CSA and muscle stiffness. Their diagnostic model, based on differences in CSA and SWE between bent and straight knee positions, achieved sensitivity and specificity of 83.3%, suggesting that SWE can be feasibly integrated into routine screening workflows. Wang et al. combined B-mode ultrasound and SWE to assess elderly T2D patients, focusing on changes in muscle thickness (MT) and the differential Young’s modulus (ΔSWE) between relaxed and contracted states [52]. Both MT and ΔSWE showed strong correlations with ASMI (r = 0.826 and 0.765, respectively; *p* < 0.01) and grip strength (r = 0.797 and 0.818, respectively; *p* < 0.01). A muscle thickness cut-off of 11.4 mm yielded an AUC of 0.952 for sarcopenia prediction, demonstrating the added diagnostic value of combining morphological and mechanical metrics.

Despite promising accuracy and strong correlations with functional and morphological indices, SWE in diabetic sarcopenia is limited by the lack of universally accepted stiffness cut-offs. While thickness thresholds (e.g., 11.4 mm in the RF) have been proposed [52], SWV values vary between populations and muscle groups due to methodological differences (probe positioning, muscle state, acquisition protocols) and patient-related factors (subcutaneous fat, edema). SWE measurements are also influenced by underlying pathological changes, particularly intramuscular fat infiltration and fibrosis, common in T2D-related myopathy, which can confound interpretation [53].

Table 2 presents a summary of studies on shear wave elastography for sarcopenia diagnosis.

### 5.2. Impact of Sarcopenia in Cirrhosis and the Emerging Utility of SWE

Sarcopenia represents a significant clinical challenge also in patients with liver disease [54,55]. Although traditional assessments focusing solely on muscle mass often fail to capture early changes in muscle quality and biomechanical properties that precede functional decline, SWE is increasingly being studied as a promising tool to address this gap [56]. A pilot study by Becchetti et al. demonstrated that 2D-SWE measurements of RF stiffness are technically feasible and highly reproducible in patients with cirrhosis [57]. Importantly, RF stiffness was found to be independent of liver function and liver frailty index (LFI), suggesting that SWE captures aspects of muscle quality that traditional liver disease severity scores and frailty measures do not reflect. While SWE stiffness values did not correlate directly with muscle size or frailty, the antero-posterior diameter of the rectus femoris muscle measured by ultrasound showed a strong inverse correlation with frailty (r = −0.578, *p* < 0.001). These results indicate that muscle stiffness and muscle size represent complementary dimensions of sarcopenia. In a related study by Enciu et al. on 43 patients with alcoholic liver disease, rectus femoris muscle stiffness measured by SWE was significantly higher in patients with alcoholic hepatitis compared to alcoholic cirrhosis (mean RF stiffness 1.78 m/s vs. 1.35 m/s, *p* = 0.001), correlating strongly with disease severity and functional performance measured by the 30-s chair stand test (*p* < 0.01) [58]. Additionally, muscle thickness and echogenicity assessed by ultrasound also correlated with clinical parameters, underscoring the value of combining SWE and morphological assessment. Although consensus on diagnostic cutoffs for SWE-derived muscle stiffness in sarcopenia is still lacking, these studies highlight SWE as a reliable, quantitative, and non-invasive tool to assess muscle biomechanical properties in cirrhosis. Its bedside applicability and reproducibility make SWE a promising complement to conventional muscle mass and frailty assessments, potentially enhancing risk stratification, monitoring, and personalized management of sarcopenia in cirrhotic patients.

While SWE is more established in assessing sarcopenia in diabetic patients, its use in cirrhotic patients remains preliminary, focusing on feasibility and reproducibility without standardized cutoffs or robust validation (Table 3). Larger, well-designed and prospective studies are needed to define the clinical role of SWE in the assessment of sarcopenia in cirrhosis.

### 5.3. Pilot Investigations of SWE in Sarcopenia Diagnosis Across Diverse Diseases

In the literature, there are only a few pilot studies investigating the use of SWE for sarcopenia in specific clinical contexts. In neurodegenerative disorders like Parkinson’s disease, SWE of the RF muscle showed decreased stiffness values correlating with reduced muscle function, and SWE was more strongly associated with physical performance than thickness or cross-sectional area [59]. This suggests that muscle elasticity is a clinically relevant parameter, potentially more directly linked to disability than morphological measurements alone. In chronic systemic conditions such as essential hypertension, SWE combined with two-dimensional ultrasound demonstrated high diagnostic accuracy for sarcopenia, with a sensitivity of 84.5% and a specificity of 90.8%, reflecting its ability to detect muscle quality deterioration alongside mass loss [60]. Similarly, in chronic obstructive pulmonary disease patients, SWE values of the RF muscle were significantly lower than healthy controls and correlated with disease severity, physical function, and inflammatory biomarkers, with SWE outperforming traditional ultrasound measurements in sarcopenia prediction [61]. In pediatric populations, SWE assessment of diaphragm thickness and stiffness revealed significantly reduced values in malnourished children compared to controls, correlating positively with anthropometric Z scores. This indicates that SWE may provide valuable insights into muscle quality and strength in pediatric sarcopenia, a novel application requiring further validation [62]. In postmenopausal women with osteosarcopenia, combining SWE shear wave velocity with ultrasound-measured rectus femoris thickness and cross-sectional area achieved a diagnostic accuracy of 88.3%, with an area under the curve (AUC) of 0.917, outperforming single parameter models. This supports SWE as a valuable addition for assessing muscle quality in metabolic bone disease contexts [63]. In chronic kidney disease (CKD) patients, a meta-analysis including 428 participants found that SWE and cross-sectional area measurements of the rectus femoris muscle had a pooled sensitivity of 95% and specificity of 73% for sarcopenia diagnosis, highlighting SWE’s utility as a rapid screening tool in this population where sarcopenia is often underdiagnosed [64]. Finally, in kidney transplant recipients, SWE revealed increased muscle elastic modulus despite decreased muscle thickness and cross-sectional area, distinguishing these patients from healthy controls and providing potential cutoff values for sarcopenia prediction [65]. Overall, these studies demonstrate SWE’s promise in capturing both muscle quantity and quality across diverse clinical settings, improving sarcopenia diagnosis beyond traditional morphometric ultrasound. Table 4 summarizes the main evidence on shear wave elastography in the diagnostic process of sarcopenia across a range of pathologies.

Table 5 summarizes the methodological characteristics of recent studies employing ultrasound elastography across various pathological conditions. Key aspects such as the studied population, measurement units, devices and manufacturers, probe frequencies, probe orientation and target muscles, patient positioning, muscle conditions during assessment, and the number of repetitions or regions of interest (ROIs) are presented to provide a comprehensive overview of the protocols used.

## 6. Conclusions

Overall, these pilot investigations highlight the promising potential of SWE to enhance sarcopenia diagnosis by capturing not only muscle quantity but also muscle quality across a variety of clinical conditions. Notably, SWE has proven to be a simple, quick, and easy-to-perform technique, making it a practical tool in daily clinical settings. By moving beyond traditional morphometric ultrasound parameters, SWE offers a more comprehensive evaluation of muscle health, which could lead to earlier detection and better tailored interventions. However, the current evidence is largely limited to small-scale, cross-sectional studies, which restricts the generalizability and strength of conclusions. To fully establish SWE’s clinical utility and integrate it into routine sarcopenia assessment and management, larger, well-designed longitudinal studies with standardized protocols are urgently needed. Such research will be essential to validate SWE’s diagnostic accuracy, define precise cut-off values, and clarify its prognostic significance, ultimately advancing personalized care for patients affected by sarcopenia in diverse disease populations.

## Figures and Tables

**Table 1:** Checklist for SWE Muscle Assessment.

Item	Notes
Patient Posture	Specify supine, prone, seated, or standing. Ensure consistent positioning across measurements.
Probe Alignment	Align parallel to muscle fibers; maintain consistent orientation throughout.
ROI Size	Record region of interest (ROI) dimensions; ensure adequate coverage of target muscle area.
Measurement Repetitions	Perform multiple measurements (e.g., 3–5 per site) to improve reliability; report mean ± SD.
Muscle State	Indicate whether the muscle is relaxed or contracted during measurement.
Reporting Template	Include: device, probe frequency, units (m/s or kPa), orientation, patient posture, muscle state, ROI size, number of repetitions, and notes on measurement quality.

**Table 2:** Shear wave elastography in diabetic sarcopenia.

Study and Authors	Muscle	SWE and Clinical Parameters	Main Findings	Diagnostic Performance
Li An et al. [49]	Medial gastrocnemius	SWV (bent and straight states), ASMI, handgrip strength	Lower SWV in sarcopenic patients (*p* < 0.01); positive correlations between SWV, ASMI, and strength	Model with BMI + ASMI + SWV: sensitivity 96.7%, specificity 82.5%, AUC 0.946
Wei et al. [50]	Gastrocnemius	Thickness, CSA, SWE, muscle mass, strength	Sarcopenic patients had lower thickness, CSA, and SWE (*p* < 0.05); moderate correlations observed	Sensitivity 81.1%, specificity 75.0%, AUC 0.800
Chen et al. [51]	Rectus femoris	CSA, SWE stiffness, differences between knee positions	Reduced CSA and stiffness in sarcopenic subjects; significant differences between bent and straight positions	Sensitivity and specificity 83.3%
Wang et al. [52]	Rectus femoris	Muscle thickness (MT), ΔSWE, ASMI, strength	Strong correlation of MT and ΔSWE with ASMI (r > 0.76) and strength (r > 0.79); thickness cut-off 11.4 mm	AUC 0.952

**Table 3:** Shear wave elastography and sarcopenia in liver diseases.

Authors	Muscle	SWE Parameter(s)	Results Summary	Diagnostic Performance
Becchetti et al. [57]	Rectus Femoris	Muscle stiffness (RFMS)	Feasible, reproducible; RFMS independent of liver function and frailty index; no correlation with frailty; muscle diameter inversely correlated with frailty (r = −0.578, *p* < 0.001)	Not established
Enciu et al. [58]	Rectus Femoris	Muscle stiffness (RFMS), thickness, echogenicity	RFMS higher in alcoholic hepatitis vs. cirrhosis (1.78 vs. 1.35 m/s, *p* = 0.001); correlated with disease severity and 30s chair stand test (*p* < 0.01)	Not established

**Table 4:** Initial evidence on SWE in the diagnostic assessment of sarcopenia in diverse pathologies.

Authors	Study Population	Study Design/Endpoint	SWE and US Parameters Measured	Key Findings
Xu Han et al. [60]	Patients with essential hypertension	Cross-sectional/Diagnosis	Muscle thickness (MT), cross-sectional area (CSA), fat layer (FL), pennation angle (PA), SWE on rectus femoris (RF) and gastrocnemius medialis (GM)	SWE combined with conventional US showed sensitivity 84.5% and specificity 90.8% for sarcopenia diagnosis
M Selçuk Sinanoğlu et al. [61]	Malnourished pediatric patients	Cross-sectional/Diagnosis	Diaphragm thickness and stiffness by SWE and US	Significant reduction in diaphragm thickness and stiffness correlated with anthropometric Z-scores
Zi-Tong Chen et al. [62]	Postmenopausal women with osteosarcopenia	Cross-sectional/Diagnosis	Rectus femoris (RF) thickness, CSA, shear wave velocity (SWV)	Combined model of RF CSA and SWV (AUC 0.917) outperformed individual parameters; sensitivity 70%, specificity 93%
Qinbo Yang et al. [63]	Chronic kidney disease (CKD) patients	Meta-analysis/Diagnosis	Rectus femoris CSA, muscle thickness (MT), SWE	High sensitivity (0.95) and moderate specificity (0.73) of CSA and SWE for sarcopenia diagnosis in CKD
Mingming Deng et al. [64]	Patients with chronic obstructive pulmonary disease (COPD)	Cross-sectional/Diagnosis	SWE mean elasticity, RF thickness, RF CSA	SWE mean elasticity had better sarcopenia prediction (AUC 0.863) than thickness and CSA; correlated with biomarkers and physical function
Yang Chen et al. [65]	Post-kidney transplant patients	Cross-sectional/Prognosis	Echo intensity, RF thickness, SWE elastic modulus	Increased echo intensity and elastic modulus in patients; SWE cutoff showed AUC ~0.84 for sarcopenia prediction

**Table 5:** Ultrasound Elastography Study Characteristics.

Study	Population	Unit of Measurement	Device/Manufacturer	Probe Frequency	Orientation/Muscle	Patient Position	Muscle Condition	Repetitions/ROI
An et al. (2025) [49]	Liver disease	SWV (m/s)	Mindray Resona I9S	4–15 MHz (linear)	Not specified/RF	Prone, feet suspended	Extended + contracted	3 per condition
Wei et al. (2023) [50]	Liver disease	Young’s modulus (kPa)	Siemens ACUSON Sequoia (ARFI)	4–9 MHz (linear)	Longitudinal/RF	Prone, legs extended	Relaxed + stretched	3 per condition
Chen et al. (2024) [51]	Liver disease	SWV (m/s)	GE LOGIQ E10 (ML6-15)	4–15 MHz (linear)	Longitudinal/RF, BB	Supine (RF) and seated (BB)	Relaxed	3 per muscle
Becchetti et al. (2023) [57]	Cirrhosis	Young’s modulus (kPa)	Aixplorer (Supersonic Imagine)	3.5–5 MHz	Transverse/RF, VM, IP	Supine, rest 7 min	Relaxed	3 per muscle
Enciu et al. (2024) [58]	Alcoholic hepatitis + cirrhosis	SWV (m/s)	Siemens Acuson S2000 (ARFI)	5–7.5 MHz (linear)	Transverse (thickness) + Long. (SWE)/RF	Supine, limbs relaxed	Relaxed	5 measures (mean + IQR)
Han et al. (2025) [60]	Hypertension (≥55 years)	Young’s modulus (kPa)	Aixplorer (Supersonic Imagine)	4–15 MHz (linear)	Transverse + Long./RF + GMM	Supine (RF) + prone (GMM)	Relaxed + contracted	3 per muscle state
Deng et al. (2022) [61]	COPD (≥40 years)	Young’s modulus (kPa)	Aixplorer (Supersonic Imagine)	4–15 MHz (linear)	Transverse/RF	Supine, dominant leg	Relaxed	3 acquisitions × 5 ROI
Sinanoğlu et al. (2024) [62]	Malnutrition (pediatric age)	Young’s modulus (kPa)	RS85 Prestige (Samsung)	LA2-14A (linear)	Transverse/Right diaphragm	Supine, right arm above head	Relaxed, end expiration	3 measures × 3 ROI (mean)
Yang et al. (2024) [64]	CKD (stages 1–5, MHD, post-Tx)	SWE (kPa), CSA (cm^2^), MT (cm)	Multiple (meta-analysis of 5 studies)	4–12 MHz (linear)	RF, transverse + longitudinal axes	Various (not unified)	Generally relaxed	Variable (study dependent)

## Data Availability

No new data were created or analyzed in this study. Data sharing is not applicable to this article.
